# Chicken eEF1α is a Critical Factor for the Polymerase Complex Activity of Very Virulent Infectious Bursal Disease Virus

**DOI:** 10.3390/v12020249

**Published:** 2020-02-23

**Authors:** Bo Yang, Nana Yan, Aijing Liu, Yue Li, Zehua Chen, Li Gao, Xiaole Qi, Yulong Gao, Changjun Liu, Yanping Zhang, Hongyu Cui, Kai Li, Qing Pan, Yongqiang Wang, Xiaomei Wang

**Affiliations:** Division of Avian Infectious Diseases, State Key Laboratory of Veterinary Biotechnology, Harbin Veterinary Research Institute, Chinese Academy of Agricultural Sciences, Harbin 150001, China; yangbo2015hsy@163.com (B.Y.); nnayan@126.com (N.Y.); laj_91@126.com (A.L.); ly1996lion@163.com (Y.L.); czh1687@163.com (Z.C.); gaoli@caas.cn (L.G.); qixiaole@caas.cn (X.Q.); gaoyulong@caas.cn (Y.G.); liuchangjun@caas.cn (C.L.); zhangyanping03@caas.cn (Y.Z.); cuihongyu@caas.cn (H.C.); likai01@caas.cn (K.L.); panqing@caas.cn (Q.P.)

**Keywords:** vvIBDV, eEF1α, VP3, polymerase

## Abstract

Infectious bursal disease (IBD) is an immunosuppressive, highly contagious, and lethal disease of young chickens caused by IBD virus (IBDV). It results in huge economic loss to the poultry industry worldwide. Infection caused by very virulent IBDV (vvIBDV) strains results in high mortality in young chicken flocks. However, the replication characteristics of vvIBDV are not well studied. Publications have shown that virus protein 3 (VP3) binds to VP1 and viral double-stranded RNA, and together they form a ribonucleoprotein complex that plays a key role in virus replication. In this study, vvIBDV VP3 was used to identify host proteins potentially involved in modulating vvIBDV replication. Chicken eukaryotic translation elongation factor 1α (cheEF1α) was chosen to further investigate effects on vvIBDV replication. By small interfering RNA-mediated *cheEF1α* knockdown, we demonstrated the possibility of significantly reducing viral polymerase activity, with a subsequent reduction in virus yields. Conversely, over-expression of *cheEF1α* significantly increased viral polymerase activity and virus replication. Further study confirmed that cheEF1α interacted only with vvIBDV VP3 but not with attenuated IBDV (aIBDV) VP3. Furthermore, the amino acids at the N- and C-termini were important in the interaction between vvIBDV VP3 and cheEF1α. Domain III was essential for interactions between cheEF1α and vvIBDV VP3. In summary, cheEF1α enhances vvIBDV replication by promoting the activity of virus polymerase. Our study indicates cheEF1α is a potential target for limiting vvIBDV infection.

## 1. Introduction

Infectious bursal disease virus (IBDV) is a non-enveloped, bi-segmented dsRNA virus, and the causative agent of infectious bursal disease (IBD), commonly known as Gumboro disease. This disease has a worldwide distribution and it is an economically important immunosuppressive disease [1,2,3]. Very virulent infectious bursal disease virus (vvIBDV) causes high mortality to young chickens aged between 3–6 weeks, and severe immunosuppression to surviving birds [3]. In China, the prevalent vvIBDV strain, which causes at least 60% mortality to flocks, remains the leading cause of economic losses for the poultry industry [4]. IBDV is especially destructive as it destroys the lymphoid organs of chickens, particularly the bursa of Fabricius (BF), which is the site where B lymphocytes maturate and differentiate. The target cells for the virus are immature B lymphocytes, and consequently, virus replication occurs mainly in the BF. Previously, mild virus strains caused only 1%–2% mortality in the United States [5]. Since the emergence of severe vvIBDV strains, the virus has spread over much of the world, and mortality has risen sharply, to as high as 100% in specific pathogen-free chickens. Today, the virus occurs in more than 50% of flocks in Europe, Asia, and Africa [6,7,8]. In China, severe vvIBDV strains have caused 60%-100% mortality in flocks over the past 20 years [4,9,10,11,12]. 

Because availability of chicken B lymphocyte cell line is restricted, most research into IBDV has been performed in chicken fibroblast cell line DF-1, using cell-adapted viruses such as classical IBDV (cIBDV) and attenuated IBDV (aIBDV). DT40 cell line, a chicken B lymphocyte cell line, is suitable for viral replication of vvIBDV in vitro, and it is an excellent tool for investigating replication cycles of vvIBDV and offering insight into vvIBDV pathogenesis. Recently, researchers employed time-course DNA microarrays to explore host gene expression patterns in DT40 cells in response to vvIBDV infection. Virus infection induced alterations in expression of some important signal transduction host genes including BCR signaling and antigen presentation [13]. Other studies used microarray to investigate gene expression profiles of chicken B cells infected with vvIBDV compared with aIBDV and found that key genes involved in B cell activation and signaling were down-regulated in infected compared to mock-infected cells, which may contribute to IBDV-mediated immunosuppression [14]. These studies provided important insights into host responses to vvIBDV infection and virus pathogenesis. However, further studies are needed to uncover host factor modulating steps of vvIBDV life cycle. 

The understanding of the replication characteristic and pathogenesis of vvIBDV is very important, and it currently remains poorly studied. The genome of vvIBDV is bi-segmented. The larger segment A has two overlapping open reading frames (ORFs). The small ORF encodes the non-structural virus protein 5 (VP5; 17 kDa) involved in viral release and cell apoptosis induced by virus [15,16,17]. The large ORF in segment A encodes a polyprotein (109 kDa) that is processed into pVP2, VP3, and VP4. Segment B encodes the RNA-dependent RNA polymerase VP1 (90 kDa) [18,19]. We became interested in the role that the virus-encoded VP3 plays in vvIBDV replication. VP3 is a multi-functional protein [20,21,22]. It has a mass of 32 kDa, and it binds to VP1 and the genomic dsRNA to form a ribonucleoprotein (RNP) complex that plays a key role in viral RNA replication. Moreover, the interaction between VP3 and VP1 facilitates RNA polymerase catalytic activation [22,23] and encapsulates VP1 and viral dsRNA into virions in the process of virus assembly [24,25]. Interactions between pVP2 and VP3 drive capsid assembly [26,27,28]. Recently, it has been reported that both VP3 and VP1 are required to translation initiation of uncapped viral dsRNA [29]. VP3 also interacts with host factors to modulate viral replication. VP3 protein competes strongly with chicken MDA5 to bind IBDV genomic dsRNA, which inhibits type I interferon production [30]. VP3 interacts with chicken ribosomal protein L18 (chRPL18) in host cells, and this enhances viral replication-associated chicken double-stranded RNA-activated protein kinase (chPKR) for suppression of type I interferon expression [31]. Interestingly, most of the research in this area has been performed on cell-adapted classical and attenuated strains, but not on the severe vvIBDV strain. Thus, our aim was to investigate host factors modulating vvIBDV replication.

In this study, we investigated host factors that might play roles in vvIBDV replication. Because VP3 performs multifunctional roles in viral replication, we investigated its interaction with host proteins identified using LC–MS/MS. Chicken eukaryotic translation elongation factor 1α (cheEF1α) was chosen for further investigation. The effect of VP3 on replication of the vvIBDV strain compared with aIBDV strain was studied. In other systems, eukaryotic translation elongation factor 1α (eEF1α) plays important roles in modulating biological functions of both host and virus [32]. For example, unique cellular and viral activities have been attributed to eEF1α in eukaryotes. Hepatitis C virus nonstructural protein 4A inhibits viral internal ribosome entry site (IRES)-mediated translation through interacting with eEF1α [33]. Human eEF1α specifically interacts with HIV-1 reverse transcriptase. Moreover, its critical role in HIV-1 reverse transcription makes eEF1α a potential anti-HIV target [34,35]. In this study, we demonstrated that cheEF1α interacts with VP3 of vvIBDV to modulate viral polymerase activity, and thereby enhances vvIBDV replication. We discuss cheEF1α as a potential target for limiting vvIBDV infection.

## 2. Materials and Methods

### 2.1. Cells, Viruses, Antibodies, and Regents

All the cells used were preserved in our lab. The DT40 (chicken B cell line) cells were kindly provided by Prof. Nair (The Pirbright Institute), and 293T was bought from ATCC. DT40 cells were maintained in RPMI-1640 Medium (R8758, Sigma, Burlington, MA, USA) supplemented with 10% fetal bovine serum (FBS), 2% chicken serum (Sigma, Burlington, MA, USA), 1% glutamine (25030-081, Gibco, Grand Island, NY, USA), 50 uM 2-mercaptoethanol, and 1% penicillin-streptomycin in a 37 °C, 5% CO_2_ incubator. 293T human embryonic kidney cells and chicken DF-1 cells were maintained in Dulbecco’s modified Eagle’s medium (DMEM; c11995500BT, Gibco, Grand Island, NY, USA) supplemented with 10% fetal bovine serum (FBS), 1% glutamine, and 1% penicillin-streptomycin in a 37 °C, 5% CO_2_ incubator. The Gx strain of vvIBDV was identified and preserved in our laboratory, and the aIBDV Gt was also preserved in our laboratory. Antibodies used in the study included mouse anti-FLAG M2 (F1804, Sigma, Burlington, MA, USA), rabbit anti-FLAG (F2555, Sigma, Burlington, MA, USA), rabbit anti-eEF1α antibody (ab175274, abcam, Cambridge, MA, USA), rabbit anti-HA (H6908, Sigma, Burlington, MA, USA), mouse-anti-HA (H9658, Sigma, Burlington, MA, USA), mouse anti-β-actin monoclonal antibody (A1978, Sigma, Burlington, MA, USA), anti-mouse IgG (whole molecule)-peroxidase antibody produced in goat (A4416, Sigma, Burlington, MA, USA), anti-rabbit IgG (whole molecule)-peroxidase antibody produced in goat (A6154, Sigma, Burlington, MA, USA), IRDye 800CW goat anti-mouse IgG H&L (926-32210, LiCor Bio-Sciences, Lincoln, NE, USA), IRDye 800CW goat anti-rabbit IgG H&L (926-32211, LiCor Bio-Sciences, Lincoln, NE, USA), goat anti-rabbit IgG H&L (Alexa Fluor 488; A-11008, Invitrogen, Grand Island, NY, USA), and goat anti-mouse IgG H&L (Alexa Fluor 546; A-11003, Invitrogen, Grand Island, NY, USA).

### 2.2. Construction of Plasmids

The eukaryotic expression plasmids based on the vvIBDV VP3 template, including pCAGGS-vvIBDV-VP3, -VP3-H28Q, -VP3-P226L, -VP3-V235A, and -VP3-A250T, or plasmids based on the aIBDV VP3 template, including pCAGGS-aIBDV-VP3, -VP3-Q28H, -VP3-L226P, -VP3-A235V, and -VP3-T250A were all constructed with a HA tag and preserved in our laboratory. Chicken eEF1α was cloned from the cDNA of DT40 cells using specific primers and was cloned into the pCAGGS plasmid with a FLAG tag. The plasmids expressing different cheEF1α domains, including cheEF1α-DI (aa: 1–245), cheEF1α-DII (aa: 246–335), cheEF1α-DIII (aa: 336–462), cheEF1α-DI+II (aa: 1–335), and cheEF1α-DII+III (aa: 246–462) were also cloned into the pCAGGS plasmid with a FLAG tag. We constructed dicistronic reporter plasmids encoding *Firefly* luciferase gene (*Fluc*) and *Renilla* luciferase gene (*Rluc*) under the control of a T7 promoter, and the vvIBDV or aIBDV 5′-UTR was inserted between *Fluc* and *Rluc* according to a previous publication [36]. For luciferase assays, the expression plasmids were generated as part of a previously established RNA polymerase II reverse genetics system preserved in our laboratory [37]. GenBank accession numbers: vvIBDV: AY444873.3; aIBDV: DQ403248.1; chicken eEF1α: L00677.1.

### 2.3. RNAi and Transfection

DT40 cells were transfected with Lonza cell line kit T (VVCA-1002, Lonza, Morristown, NJ, USA) according to the manufacturer’s instructions. Three siRNAs specifically targeting the eEF1α mRNA of Gallus were designed by the GenePharma Company (Suzhou, China) to study viral replication. siRNA sequences for knockdown of eEF1α in DT40 cells included RNAi#1 (sense, 5′-GGCCAAAUCAGUGCUGGUUTT-3′), RNAi#2 (sense, 5′-GGCACAGAGACUUCAUUAATT-3′), RNAi#3 (sense, GCAUCGACAAGAGGACCAUTT), and negative siRNA control (sense, 5′-UUCUCCGAACGUGUCACGUTT-3′). siRNA transfection in 293T cells included siRNA (Sigma, Shanghai, China) targeting eEF1α (siRNA ID: SASI_Hs02_00331771 and 00331772) and control siRNA (SIC001), and was performed using RNAiMAX (13778150, Invitrogen, Grand Island, NY, USA) according to the manufacturer’s instructions. Double transfections were performed at 24 h intervals. Then, 24 h after the second transfection, cells were harvested for further analysis. The siRNA with the highest knockdown efficiency was chosen for evaluating the influence of eEF1α on vvIBDV replication. For the replication study, siRNA-transfected cells were infected with the vvIBDV Gx strain at a multiplicity of infection (MOI) of 1 and cultured for an additional 72 h. Similarly, the cells of over-expression of eEF1α were infected with Gx strain at a MOI of 1 and cultured for an additional 72 h. Then, supernatants and cell cultures were collected to detect viral titres and proteins of vvIBDV. 

### 2.4. Virus Infection and ELD_50_ Titration

For viral infection, the DT40 cells were counted by cell counter, and appropriately dilute viruses were incubated with cells for 4 h in a 41 °C, 5% CO_2_ incubator. Subsequently, viral inoculum was removed and cells were maintained with 1640 complete medium at 41 °C until collection. The chicken embryos were used to titrate infectious progeny viruses after various treatments. Infected cell supernatants were harvested at specific time points after infection, and the titres of infectious viral progenies presented in the supernatants were determined in terms of 50% embryo lethal dose (ELD_50_)/100 μL using the Reed–Muench formula. All experiments were repeated three times, and the means and standard deviations were calculated. All chicken embryo experiments were approved by the Committee on the Ethics of Animal Experiments at the Harbin Veterinary Research Institute (Harbin, China), Chinese Academy of Agricultural Sciences, and performed in accordance with the Guidelines for Experimental Animals of the Ministry of Science and Technology (Beijing, China).

### 2.5. Immunoprecipitation, SDS-PAGE, and Silver Staining 

For immunoprecipitation, DF-1 cells were seeded on 6-well plates and cultured until cells were at 80% confluence before being transfected with pCAGGS-HA-VP3 or pCAGGS by TransIT-X2 Transfection System (Mirus, Madison, WI, USA). Next, 36 h after transfection, transfected cells were lysed in cell lysis buffer for Western blot and IP (P0013, Beyotime, Shanghai China). Then, supernatants were obtained by centrifuging and were incubated with 20 μL monoclonal anti-HA−agarose antibody produced in mouse (A2095, Sigma, Burlington, MA, USA) at 4 °C for 6–8 h or overnight. After that, beads were washed five times with PBS, then boiled with 5 × SDS loading buffer (P0015L, Beyotime, Shanghai, China) for 10 min. Subsequently, the samples were fractionated by electrophoresis on 12% SDS-polyacrylamide gel and stained by Fast Silver Stain Kit (P0017, Beyotime, Shanghai, China). In brief, the gel was fixed for 1 h and washed by 30% ethyl alcohol and ultrapure water; then, the gel was sensitized and washed was twice by ultrapure water, and the gel was then stained with silver buffer and washed for no more than 1.5 min; finally, the gel was developed until it showed distinct protein bands, wherein the developing was stopped and the discrepant bands were cut to perform LC–MS/MS analysis (HOOGEN BIOTECH, Shanghai, China).

### 2.6. Co-Immunoprecipitation and Western Blot 

First, 293T cells were seeded on 6-well plates and cultured until cells were at 80% confluence before being transfected with pCAGGS-HA-vvIBDV-VP3, or pCAGGS-HA-aIBDV-VP3, and/or pCAGGS-Flag-eEF1α by TransIT-X2 Transfection System (Mirus, Madison, WI, USA). The transfections of viral VP3 mutant plasmids or eEF1α different domain plasmids were performed similarly. Next, 36 h after transfection, transfected cells were lysed in cell lysis buffer for Western blot and IP (P0013, Beyotime, Shanghai, China). Then, supernatants were obtained by centrifuging and were incubated with 20 μL anti-FLAG M2 affinity gel (A2220, Sigma, Burlington, MA, USA) at 4 °C for 6–8 h or overnight. At the same time, we performed co-immunoprecipitation assays in the opposite direction. The supernatants were incubated with 1 μg anti-HA rabbit mAb at 4 °C for 6–8 h. After incubation with antibody, 25 μL protein A/G-agarose (A10001, Abmart, Shanghai, China) was added, and the samples were incubated at 4 °C overnight. Beads were washed five times with PBS, then boiled with 5 × SDS loading buffer (P0015L, Beyotime, Shanghai, China) for 10 min. Subsequently, the samples were fractionated by electrophoresis on 12% SDS-polyacrylamide gels, and resolved proteins were transferred onto nitrocellulose membranes. After blocking with 5% skim milk, the membranes were incubated with rabbit anti-FLAG and mouse anti-HA antibodies; then, Western blot analysis was performed with corresponding secondary antibodies.

### 2.7. RNA Extraction and RT-qPCR

Total RNA was extracted using a RNeasy Mini Kit (74106, QIAGEN, Germany), and 2 μg of RNA was reverse-transcribed into cDNA using a ReverTra Ace qPCR RT Master Mix with gDNA remover (FSQ301, TOYOBO, Shanghai, China) in a 10-μL reaction mixture. cDNA was analysed using RT-qPCR using the Flourescent Quantitative PCR Instrument (Mx 3005P, Aligent, Palo Alto, CA, USA). Specific primers and TaqMan probes for chicken 28S, as well as IBDV VP5, were synthesized by Invitrogen (Shanghai, China). RT-qPCR was performed with the following cycling conditions: 95 °C for 1 min for initial denaturation, followed by 40 cycles of 95 °C for 15 s for denaturation, 60 °C for 1 min, and collection of PCR product signals. All controls and infected samples were examined in triplicate on the same plate. cDNA quantities were normalised to 28S cDNA quantities measured from the same samples.

### 2.8. Confocal Microscopy

293T cells were transfected with pCAGGS-Flag-eEF1α and/or pCAGGS-HA-VP3 for 36 h. Then, the cells were washed with PBS three times and fixed with 4% formaldehyde for 30 min, followed by permeabilization with 0.1% Triton X-100 in PBS for 15 min. Samples were rinsed with PBS and blocked with 5% skim milk in PBS at 37 °C for 2 h before being incubated with mouse anti-HA (1:200) and/or rabbit anti-FLAG (1:200) diluted in PBS for 2 h at 37 °C. Then, cells were washed three times with PBS and incubated with the secondary antibodies Alexa 488 anti-rabbit and Alexa 546 anti-mouse (1:500). Finally, cells were stained with 4′6-diamidino-2-phenylindole (DAPI) at room temperature for 15 min (C1005, Beyotime, Shanghai, China).

### 2.9. IBDV Minigenome System for Detection of Polymerase Activity

Human 293T cells were seeded onto 12-well plates. After 24 h, cells were transfected with the expression plasmids (1 μg of pCAGGS-Flag-eEF1α or empty vector), 200 ng of plasmid pRL-TK (pRL vectors were wild-type *Renilla* luciferase (*Rluc*) control reporter vectors, and pRL-TK had an HSV-thymidine kinase promoter, bought from Promega), and 1 μg of each of the following RNA polymerase plasmids: pCAGGS-vvIBDV-VP3, virus segment B, and luciferase reporter genome pCAGGS-Luc-vvIBDV using TransIT-X2 Transfection System (Mirus, Madison, WI, USA). The pRL-TK vector was used as an internal control to correct for differences in both transfection and harvest efficiencies. Then, 36 h after transfection, cells were lysed and analyzed with a dual-luciferase reporter assay kit according to the instructions of the manufacturer (E1960, Promega, Madison, WI, USA). siRNA targeting eEF1α of *Homo sapiens* (siRNA ID: SASI_Hs02_00331771 and 00331772, Sigma, Shanghai, China) and control siRNA (SIC001) were transfected into 293T cells using RNAiMAX according to the manufacturer’s instructions. Double-transfections were performed at 24 h intervals; meanwhile, cells were transfected with vvIBDV minigenome system as described above. Finally, 36 h after transfection, cells were harvested for analysis of polymerase activity.

### 2.10. Statistical Analysis

Data are presented as means ± standard deviations (SD). In survival curve, the significance of the variability between different groups was determined by the GraphPad Prism software (version 6.0). In other experiments, the statistical analyses were performed with the unpaired *t*-test. *p* < 0.05 was considered significant and marked with one asterisk (*), and *p* < 0.01 is marked with two asterisks (**).

## 3. Results

### 3.1. Host Proteins Immunoprecipitated with vvIBDV VP3

**Figure 1:**
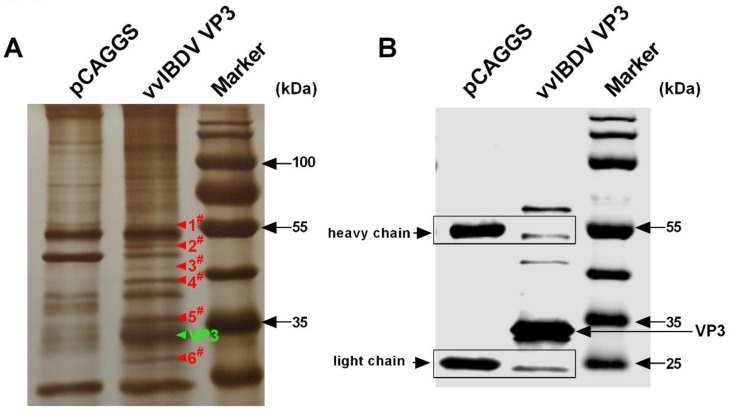
Very virulent infectious bursal disease virus (vvIBDV) virus protein 3 (VP3) was used to fish potential associated host factors. (**A**) Six different gel bands were detected in vvIBDV VP3 immunoprecipitated cell lysates compared with pCAGGS vector control sample. DF-1 cells were transfected with eukaryotic expression plasmids harboring the HA-tagged vvIBDV VP3 or empty vector pCAGGS. Cells were lysed with commercial IP and WB lysis buffer after 36 h post-transfection, and cell lysates were incubated with anti-HA protein A/G at 4 °C overnight. Then, the immunoprecipitated protein A/G were collected for SDS-PAGE, and the different gel bands were sent for LC–MS/MS identification. Meanwhile, the expression of transfected proteins was confirmed by Western blotting with anti-HA antibody, and results were shown in (**B**).

Viral VP3 is a multifunctional “scaffold” protein and plays important roles in the virus life cycle [20,22,29,38]. Thus, it is identified as a potential target to disrupt virus–host interactions [30,31,39]. However, there are few studies based on vvIBDV VP3 that investigate replication characteristics of vvIBDV. In order to explore potential host factors associated with the influence of VP3 on virus replication, we immunoprecipitated the host factors associated with vvIBDV VP3. As shown in silver-stained gel (Figure 1A), there are many different bands that showed up in the vvIBDV VP3 lane compared to the pCAGGS negative control. The six most pronounced bands were identified by LC–MS/MS, and a band corresponding to vvIBDV VP3 (on the basis of its predicted molecular weight) was present. Moreover, the expression of vvIBDV VP3 was confirmed by Western blotting (Figure 1B). The LC–MS/MS analysis of bands 1 to 6 showed that hundreds of proteins were identified in each protein band. On the basis of the unique peptide count of no less than one (UniquePepCount ≥1), the six bands had 196, 187, 128, 147, 119, or 101 candidate host proteins, respectively. On the basis of the molecular weight of each candidate host protein, we identified potential host proteins (Table S1). Ribosomal proteins and translation-related proteins were identified among the potential host proteins. Interestingly, “elongation factor 1 alpha” (reference F1N9H4 in the TrEMBL database) was identified in protein band 1, and “eukaryotic translation elongation factor 1” (Q6EE30) was identified in protein band 2. Considering the multiple functions that eukaryotic translation elongation factor 1 alpha performs in cellular and viral biological processes, the chicken eukaryotic translation elongation factor 1 alpha (cheEF1α) was chosen for further investigation.

### 3.2. cheEF1α is Critical for vvIBDV Replication

**Figure 2:**
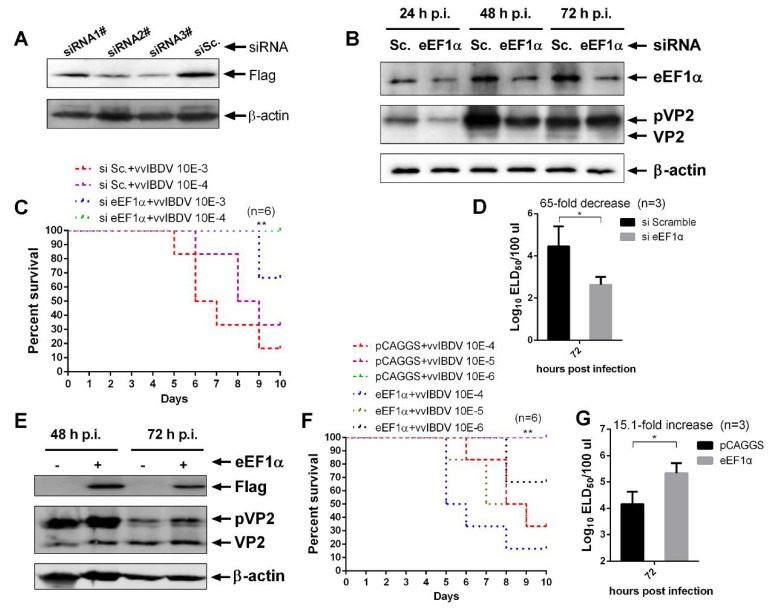
Chicken eukaryotic translation elongation factor 1α (cheEF1α) promoted the replication of vvIBDV. (**A**) Effects of siRNAs on the cheEF1α expression. 293T cells were transfected with Flag-tagged cheEF1α accompanied with siRNAs (RNAi#1, RNAi#2, or RNAi#3) or scrambled siRNA. Cell lysates were harvested 48 h after transfection and tested by Western blotting with anti-Flag antibody. Endogenous β-actin expression was used as an internal control. (**B**) Yields of viral proteins in cheEF1α knockdown cells. Viral proteins loads were analyzed by Western blotting at 24 h, 48 h, and 72 h post-infection (p.i.) with vvIBDV Gx at multiplicity of infection (MOI) = 1 compared the potent cheEF1α siRNA treatment to scrambled siRNA treatment. The amount of cheEF1α indicated the efficiency of siRNA silencing and β-actin was used as the loading control. (**C**) Typical survival curve of chicken embryos infected with extracellular progeny virus in cheEF1α knockdown cells. Progeny viruses in extracellular of cells with or without cheEF1α knockdown were harvested at 72 h p.i., and chicken embryos were infected with 10-fold diluted progeny viruses, with the number of deaths for 9 days post-infection being recorded. One of three independent tests was shown as survival curve, and these three independent tests of 50% embryo lethal dose (ELD_50_) were summarized in (**D**). (**E**) Yields of viral proteins in cheEF1α over-expression cells. Viral protein loads were analyzed by Western blotting at 48 h and 72 h p.i. with vvIBDV Gx at MOI = 1 with or without cheEF1α over-expression. (**F**) Typical survival curve of chicken embryos infected with extracellular progeny virus in cheEF1α over-expression cells. The progeny virus titers based on 50% embryo lethal dose (ELD_50_) are summarized in (**G**). Error bars and mean ± SD were calculated on the basis of three independent experiments. The statistical analysis of survival curve was analyzed with GraphPad Prism 6 software, and the other statistical analysis was performed with *t*-tests considered significant with one asterisk as *p* < 0.05 and two asterisks as *p* < 0.01.

eEF1α is important in polypeptide synthesis and other functions in cellular and viral activities [32]. In order to assess the effect of cheEF1α on vvIBDV replication, we used siRNA knockdown or over-expression of cheEF1α to evaluate the amount of viral proteins bound intracellularly using Western blotting, and we used 50% embryo lethal dose (ELD_50_) to evaluate progeny virus titres extracellularly. We first scanned the efficiency of siRNAs targeting *cheEF1α*, and two pairs of siRNAs effectively down-regulated *cheEF1α* expression (Figure 2A). Expression of *cheEF1α* was silenced in DT40 cells, and cells were infected with 1 MOI (multiplicity of infection) of vvIBDV. At 24 h post-infection (h p.i.), 48 h p.i., and 72 h p.i, the amounts of viral protein and progeny virus titres were determined. As shown in Figure 2B, *cheEF1α* expression was effectively down-regulated and viral proteins pVP2 and VP2 decreased over the 24–72 h p.i. period, and the typical chicken embryo survival curve showed that progeny virus titres were significantly reduced at 72 h p.i. (Figure 2C). On the basis of three independent tests, the progeny virus titres sharply decreased 65-fold extracellularly (Figure 2D), which indicated that down-regulation of *cheEF1α* significantly inhibited replication of vvIBDV. Over-expression of *cheEF1α* resulted in an increase of viral protein yields at 48–72 h p.i., a significant change in the embryo survival curve and a 15.1-fold increase in extracellular progeny virus titres (Figure 2E-G). Taken together, these results show that cheEF1α is critical to the replication of vvIBDV.

### 3.3. cheEF1α Interacts with vvIBDV VP3, but not with aIBDV VP3

**Figure 3:**
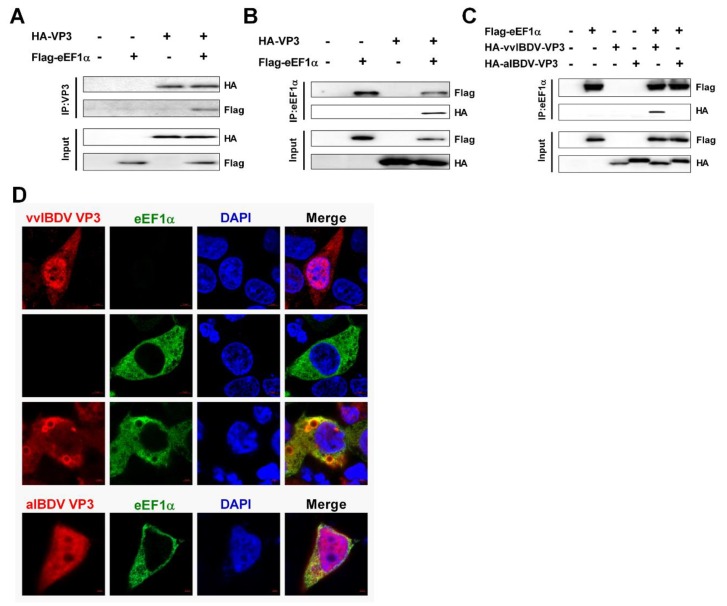
cheEF1α interacts with vvIBDV VP3, but not aIBDV. VP3 (**A**) 293T cells were transfected with HA-vvIBDV VP3 and Flag-cheEF1α expression plasmids. Cell lysates were prepared at 36 h post transfection and immunoprecipitated with anti-HA antibody and immunoblotted with anti-Flag or anti-HA antibodies. (**B**) 293T cells were transfected as in (A). Cell lysates were prepared at 36 hours post transfection and immunoprecipitated with anti-Flag antibody and immunoblotted with anti-Flag or anti-HA antibodies. (**C**) 293T cells were transfected with HA-vvIBDV VP3 and Flag-cheEF1α or HA-aIBDV VP3 and Flag-cheEF1α expression plasmids. Cell lysates were prepared at 36 h post transfection and immunoprecipitated with anti-Flag antibody and immunoblotted with anti-Flag or anti-HA antibodies. (**D**) 293T cells were transfected with HA-vvIBDV VP3 and/or Flag-cheEF1α for 36 h and then fixed and processed for dual-immunostaining. Cell nuclei were counterstained with 4′6-diamidino-2-phenylindole (DAPI) (blue). VP3 (red) and cheEF1α (green) proteins were visualized by labelling with anti-HA and/or anti-Flag antibodies, respectively, and were observed using confocal microscopy. Scale bars were marked down right corner. VP3 staining is shown in red, and cheEF1α staining is shown in green; merged images are shown with areas of co-localization in yellow. Each co-immunoprecipitation experiment was repeated three times.

In order to assess whether cheEF1α interacts with vvIBDV VP3 or aIBDV VP3, we performed a co-immunoprecipitation assay based on eukaryotic expression plasmids harboring *cheEF1α* linked to a Flag tag, and vvIBDV VP3 or aIBDV VP3 linked to a HA tag. The proteins expressed correctly, and the vvIBDV VP3 co-immunoprecipitated with the host factor cheEF1α (Figure 3A). These results were confirmed in the opposite direction (Figure 3B), and the host factor cheEF1α could also co-immunoprecipitate with vvIBDV VP3. Thus, it was clear that cheEF1α interacts with vvIBDV VP3 in both directions. In contrast, there was no interaction between cheEF1α and aIBDV VP3, irrespective whether cheEF1α or aIBDV VP3 were used to co-immunoprecipitate each other (data not shown). Moreover, in order to further confirm that cheEF1α interacts with vvIBDV VP3, but not with aIBDV VP3, we tested the co-immunopreciptation between cheEF1α and vvIBDV VP3, or cheEF1α with aIBDV VP3, in the same experiment. The results further confirmed that cheEF1α interacts with vvIBDV VP3, but not with aIBDV VP3 (Figure 3C). Confocal microscopy showed that vvIBDV VP3 was localized both in the cytoplasm and the nucleus when it was expressed alone in 293T cells, and expressed cheEF1α was localized in the cytoplasm. When vvIBDV VP3 was co-expressed with cheEF1α in the same cells, they were mainly localized in the cytoplasm and there was an obvious partial co-localisation. Interestingly, some vacuoles appeared in co-expressed cells, and the vvIBDV VP3 and cheEF1α co-localized on the surface of these vacuoles. However, there was no obvious co-localization in cells co-expressed with aIBDV VP3 and cheEF1α, and no vacuoles appeared (Figure 3D). Taken together, results indicated that cheEF1α interacts with vvIBDV VP3 in the cytoplasm, but it does not interact with aIBDV VP3.

### 3.4. Both N- and C-Terminal Amino Acids of VP3 Are Essential in the Interaction Between vvIBDV VP3 and cheEF1α

**Figure 4:**
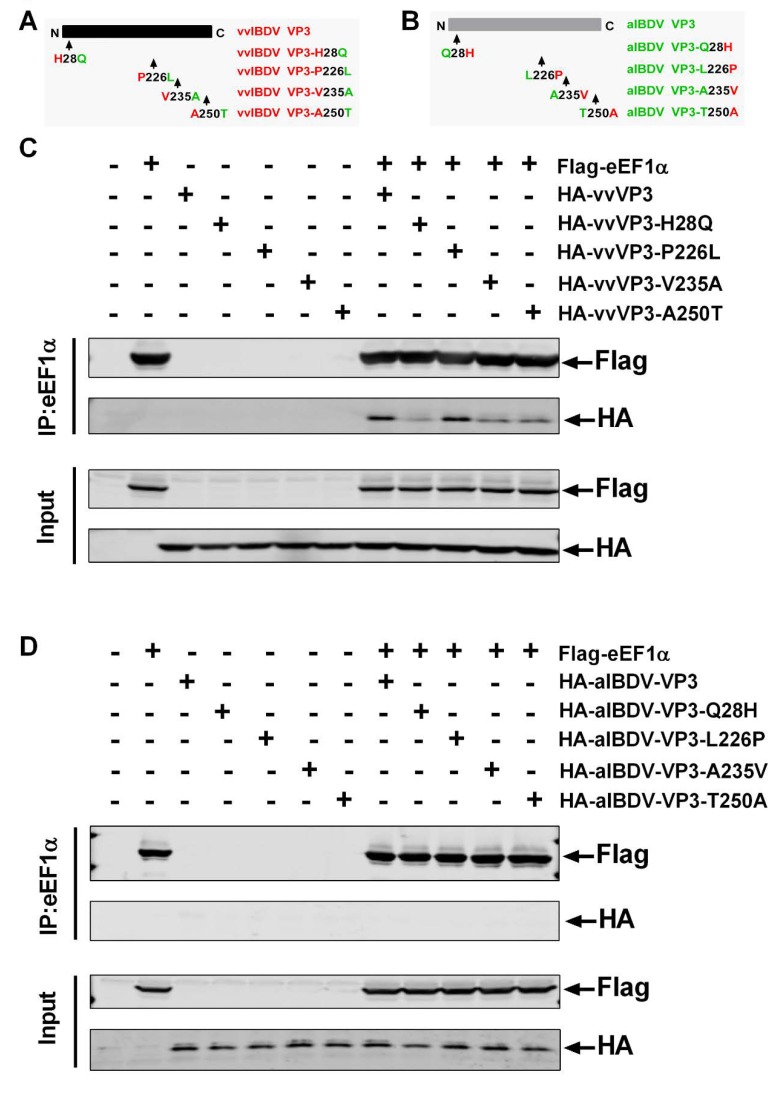
Both N- and C-terminal amino acids of VP3 are essential in the interaction between vvIBDV VP3 and cheEF1α. (**A**) Four mutant plasmids with only one amino acid mutation including vvIBDV-VP3-H28Q, -VP3-P226L, -VP3-V235A, and -VP3-A250T were constructed using vvIBDV VP3 as the template. (**B**) Four mutant plasmids aIBDV-VP3-Q28H, -VP3-L226P, -VP3-A235V, and -VP3-T250A with one amino acid substitution were constructed with attenuated VP3 as the template. (**C**) 293T cells were transfected expression plasmids with Flag-cheEF1α and HA-vvIBDV VP3, Flag-cheEF1α and vvIBDV-VP3-H28Q, Flag-cheEF1α and vvIBDV-VP3-P226L, Flag-cheEF1α and vvIBDV-VP3-V235A, or Flag-cheEF1α and vvIBDV-VP3-A250T. Cell lysates were prepared at 36 h post-transfection and immunoprecipitated with anti-Flag antibody and immunoblotted with anti-Flag or anti-HA antibodies. (**D**) 293T cells were transfected expression plasmids with Flag-cheEF1α and HA-aIBDV VP3, Flag-cheEF1α and aIBDV-VP3-Q28H, Flag-cheEF1α and aIBDV-VP3-L226P, Flag-cheEF1α and aIBDV-VP3-A235V, or Flag-cheEF1α and aIBDV-VP3-T250A. Cell lysates were prepared at 36 h post-transfection and immunoprecipitated with anti-Flag antibody and immunoblotted with anti-Flag or anti-HA antibodies. Each co-immunoprecipitation experiment was repeated three times.

Alignment of deduced amino acid sequences of VP3 proteins of vvIBDV strain Gx and attenuated strain Gt revealed that there are four amino acid mutations in VP3, one of which was located at the N-terminal and other three were located at the C-terminal of the protein [10]. We used vvIBDV VP3 as the template to construct four mutant plasmids, each with one amino acid mutation. These were vvIBDV-VP3-H28Q, -VP3-P226L, -VP3-V235A, and -VP3-A250T (Figure 4A). The interaction between cheEF1α and vvIBDV VP3 or VP3 mutants was tested by CO-IP assay. The results showed that cheEF1α co-precipitated with vvIBDV VP3. The vvIBDV-VP3 and the mutant vvIBDV-VP3-P226L were co-precipitated by cheEF1α in similar amounts. However, mutants vvIBDV-VP3-V235A and vvIBDV-VP3-A250T were co-precipitated by cheEF1α in smaller amounts, and co-precipitation of vvIBDV-VP3-H28Q was significantly reduced (Figure 4C). These findings indicated that amino acid site 28 at the N-terminal of vvIBDV VP3 was critical in the interaction between vvIBDV VP3 and host factor cheEF1α, and that sites 235 and 250 at the C-terminal were also involved in this interaction. Four mutated plasmids: aIBDV-VP3-Q28H, -VP3-L226P, -VP3-A235V, and -VP3-T250A, were constructed by mutagenesis of one amino acid with the attenuated VP3 as the template (Figure 4B). As shown in Figure 4D, neither the attenuated VP3 nor the mutants with only one amino acid mutation, including aIBDV-VP3-Q28H, -VP3-L226P, -VP3-A235V, or -VP3-T250A, were co-precipitated by cheEF1α. This also indicated that there was no interaction between aIBDV VP3 and cheEF1α, and a single amino acid mutation could not change this result. Taken together, our results indicated that specific amino acids at the N- and C-terminal are important for the interaction between vvIBDV VP3 and cheEF1α. 

### 3.5. The Third Domain of cheEF1α Interacted with vvIBDV VP3

**Figure 5:**
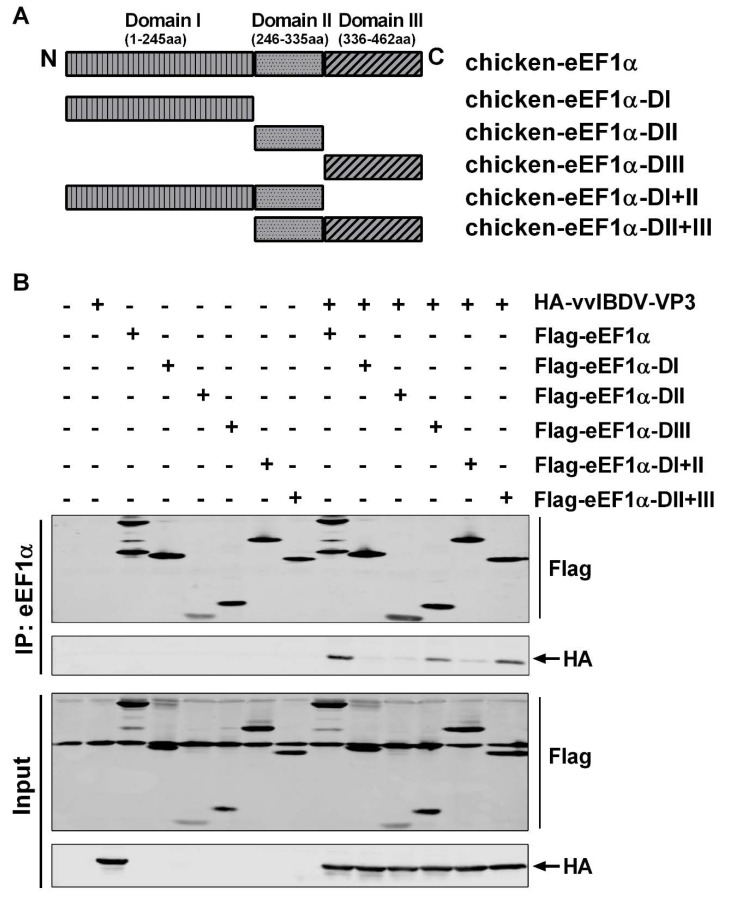
The third domain of cheEF1α interacted with vvIBDV VP3. (**A**) The expression plasmids harboring different domains of cheEF1α were depicted as cheEF1α-DI, cheEF1α-DII, cheEF1α-DIII, cheEF1α-DI+II, and cheEF1α-DII+III. (**B**) 293T cells were transfected expression plasmids with HA-vvIBDV VP3 and Flag-cheEF1α, HA-vvIBDV VP3 and Flag-cheEF1α-DI, HA-vvIBDV VP3 and Flag-cheEF1α-DII, HA-vvIBDV VP3 and Flag-cheEF1α-DIII, HA-vvIBDV VP3 and Flag-cheEF1α-DI+II, or HA-vvIBDV VP3 and Flag-cheEF1α-DII+III. Cell lysates were prepared at 36 h post transfection and immunoprecipitated with anti-Flag antibody and immunoblotted with anti-Flag or anti-HA antibodies. Each co-immunoprecipitation experiment was repeated three times.

Three well-defined structural domains are implicated in different aspects of cheEF1α function. Domain I binds GTP [32], domain II is involved in the binding of the CCA-aminoacyl end of the tRNA [40], domains I and II bind the eEF1β complex [32], and domains III and I interact with actin [41,42]. In order to investigate which domain of cheEF1α is essential for the interaction with vvIBDV VP3, we constructed plasmids expressing different domains, including cheEF1α-DI (aa: 1–245), cheEF1α-DII (aa: 246–335), cheEF1α-DIII (aa: 336–462), cheEF1α-DI+II (aa: 1–335), and cheEF1α-DII+III (aa: 246–462) (Figure 5A). The interaction was observed by the co-immunoprecipitation test with vvIBDV VP3 compared to the full-length cheEF1α. The experiment showed that all domains of cheEF1α were expressed normally, and the full-length cheEF1α, as a positive control, could co-precipitate vvIBDV VP3. Compared with the full-length protein, only domain III or domain II + III co-precipitated VP3, whereas domains I or II did not (Figure 5B). This indicates that domain III of cheEF1α is essential for the interaction with vvIBDV VP3.

### 3.6. cheEF1α Promotes the Activity of vvIBDV Polymerase

**Figure 6:**
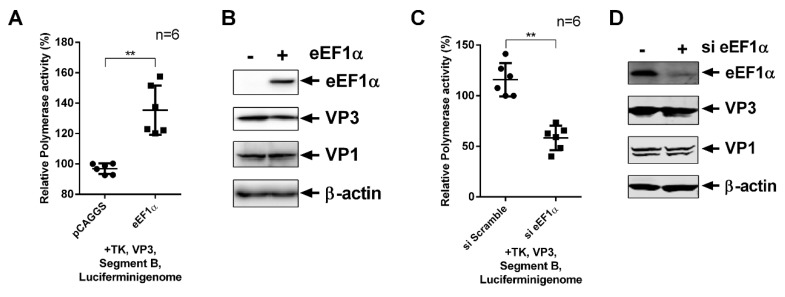
cheEF1α enhances the activity of vvIBDV polymerase. (**A**,**B**) Effects of cheEF1α over-expression on virus polymerase activity. The polymerase activity assays were performed in 293T cells expressing vvIBDV segment B-driven luciferase minigenome system, accompanied with VP3, segment B, TK, and cheEF1α or empty vector pCAGGS. VP1, VP3, and cheEF1α protein expressions were analyzed by Western blotting with β-actin as a loading control. (**C**,**D**) Effects of eEF1α knockdown on virus polymerase activity. 293T cells were first transfected with commercial competent eEF1α siRNAs or scrambled control siRNAs. Polymerase activity assays were performed at 36 h post-second transfection with viral polymerase system as described in (A). VP1, VP3, and eEF1α protein expressions were analyzed by Western blotting with β-actin as a loading control. Error bars, mean ± SD were calculated on the basis of three independent experiments. Statistical analysis was performed with the *t*-test with significance shown by two asterisks as *p* < 0.01.

We investigated the mechanism underlying the modulation on virus replication of host factor eEF1α, which is responsible for the delivery of aminoacyl-tRNAs to the ribosome, aside from initiator and selenocysteine tRNAs. We constructed dicistronic reporter plasmids encoding *Firefly* luciferase gene (*Fluc*) and *Renilla* luciferase gene (*Rluc*) under the control of a T7 promoter, and the vvIBDV or aIBDV 5′-UTR was inserted between *Fluc* and *Rluc* (data not shown). In some RNA viruses, such as classical swine fever virus (CSFV) and hepatitis C virus (HCV), the 5′-UTR harbors an internal ribosome entry site (IRES), which is an important target site for host factors to modulate the virus replication [33,43]. However, previous studies did not elucidate whether the 5′-UTR of IBDV possesses an IRES. In our study, we demonstrated that neither vvIBDV nor aIBDV 5′-UTR perform the function of IRES (data not shown). The function of cheEF1α on virus polymerase activity was assessed on the basis of our mini-genome system reported previously [37,44]. Over-expression of cheEF1α significantly increased viral polymerase activity by about 40%, without increases of expression of viral proteins VP1 and VP3. Moreover, knockdown of *eEF1α* by siRNAs, as described in previous studies [35], significantly inhibited viral polymerase activity by about 50% without decreasing expression of viral proteins VP1 and VP3 (Figure 6A–D). Taken together, our results show that cheEF1α promotes activity of the virus polymerase to enhance vvIBDV replication.

## 4. Discussion

In this study, vvIBDV VP3 was used to identify potential host factors in an immunoprecipitation assay. We found that chicken eukaryotic translation elongation factor 1α (cheEF1α) likely plays important roles in virus replication associated with VP3. We further demonstrated that cheEF1α only interacts with vvIBDV VP3, but not with aIBDV VP3. We also provided evidence that domain III of cheEF1α is essential for interaction with vvIBDV VP3. Knocking down expression of *cheEF1α* significantly inhibited vvIBDV replication, whereas its over-expression increased virus replication. Moreover, cheEF1α enhanced viral polymerase activity to promote virus replication. Similar interactions between viral polymerases and host elongation factors were described in vesicular stomatitis virus [45], West Nile virus [46], influenza virus [47], and tobacco mosaic virus [48].

Our study identified cheEF1α as an important host factor in facilitating vvIBDV replication, and this discovery suggests cheEF1α is a potential host target that could be used to restrict vvIBDV replication to improve chicken breeds. eEF1α is important in other virus replication systems. In soybean mosaic virus (SMV), a member of the ssRNA plant-infecting viruses of the large genus *Potyvirus*, protein 3 (P3) is a viral virulence determinant that interacts with the soybean (*Glycine max*) homologue of cheEF1α, GmeEF1A [49]. The SMV P3 targeted GmeEF1A, resulting in the unfolded protein response, which in turn facilitated SMV replication. The interaction of P3 and GmEF1A also promoted nuclear localization in each other, which also occurred in human EF1A and the HIV Nef protein [50]. As occurring in vvIBDV and cheEF1α, SMV replication was strongly inhibited when expression of *GmeEF1A* was knocked down. Of significance to our study is that knockdown of *GmeEF1A* did not alter morphology in soybean, and this finding lends support for *cheEF1α* as a target in developing chicken lines resistant to vvIBDV. Potentially, this could be achieved by screening for natural mutants, or by targeted editing of the *cheEF1α* gene in chicken cell lines using CRISPR/Cas9 or similar technologies. 

Understanding the basis of resistance to viruses is absolutely critical in developing improved virus-resistant lines for the poultry industry. Another important virus of chickens is avian influenza virus. Mx proteins are large GTPases [51] that interfere with the replication of avian influenza virus (and other influenza viruses) by inhibiting activity of viral polymerases [52]. Different Mx alleles confer resistance or susceptibility to avian influenza virus replication [53], and thus acknowledgement of their frequencies in wild and domestic chickens is important when considering potential breeding lines for improvement of modern commercial flocks. For example, analysis of natural Mx alleles revealed a high frequency of the susceptibility allele in broiler flocks compared to egg-laying flocks and ancestral breeds [54]. Similar studies should be conducted for alleles of *cheEF1α* to determine if they confer resistance or susceptibility to vvIBDV.

Infection with vvIBDV strains causes high mortality in young chickens. However, replication characteristics of vvIBDV are not well known. VP3 is a multifunctional protein in the virus life cycle, and VP3 interacts directly with VP1 (RdRp) and viral dsRNA to constitute the ribonucleoprotein (RNP) complex that executes the virus RNA replication and transcription. VP3 also performs important roles in virus assembly, which facilitates VP2 to construct the capsid and package viral dsRNA and proteins into virions. Thus, we chose VP3 protein to fish for host factors involved in vvIBDV replication. Our work revealed that cheEF1α promotes vvIBDV replication, and evidence for this claim was gained from knockdown and over-expression assays. 

To explore specific mechanisms, we analyzed whether cheEF1α modulated vvIBDV translation. eEF1α is the main factor responsible for the delivery of all aminoacyl-tRNAs to the ribosome, except for initiator and selenocysteine tRNAs [32]. In some RNA viruses, such as classical swine fever virus (CSFV) and hepatitis C virus (HCV), the 5′-UTR harbors an internal ribosome entry site (IRES), which is an important target site for host factors in order to modulate the virus replication [33,43]. However, previous studies have not indicated clearly whether the 5′-UTR of IBDV possesses a functional IRES. In this study, we demonstrated that neither vvIBDV nor aIBDV 5′-UTR perform the function of IRES, and thus cheEF1α could not modulate the virus replication in this way. eEF1α is involved in many important biological processes, including virus–host interactions, subcellular organization, and the integration of key cellular pathways [32]. David Harrich and co-workers demonstrated that association between eEF1α and reverse transcription complex (RTC) occurs through a strong and direct interaction between the subunit eEF1α and reverse transcriptase (RT). The HIV-1 RTC and the association is important for late steps of reverse transcription and virus replication, thus the RT-eEF1α interaction is a potential drug target [34,35]. Moreover, this team further demonstrated that HIV-1 uncoating and reverse transcription require eEF1α binding to surface-exposed acidic residues of the RT thumb domain [55]. Taken together, eEF1α might be an ideal host target to restrict HIV-1 reverse transcription and inhibit virus replication. In our study, we demonstrated that cheEF1α only interacts with vvIBDV VP3 but not with aIBDV, and both N- and C-terminal amino acids of VP3 are essential for the interaction of vvIBDV VP3 with cheEF1α. Because VP3 is a multifunctional protein in the virus life cycle and a main component of viral RNPs associated with VP1 and virus dsRNA [22], we speculated that eEF1a modulates virus replicase complex activity to effect virus replication. cheEF1α over-expression indeed upregulated polymerase activity of vvIBDV. Similarly, polymerase activity was down-regulated when cheEF1α was downregulated by siRNA, and there was no influence on the amounts of viral VP3 and VP1 present. cheEF1α was also observed as facilitating vvIBDV VP3 export into the cytoplasm from the nucleus, which is an eEF1α non-canonical function in nuclear export events. Interestingly, vacuoles appeared in the cytoplasm, and these had strong co-localization signals on membrane surfaces. This finding supports findings by others that these cellular membranes are important for the virus “replication factory” to bind to [56,57]. A recent study proved that IBDV hijacks endosomal membranes as the scaffolding structure for viral replication [58]. Taken together, our research shows that cheEF1α facilitates vvIBDV VP3 nuclear export and enhances virus polymerase activity to promote vvIBDV replication.

## Supplementary Materials

Supplementary materials can be found at https://www.mdpi.com/1999-4915/12/2/249/s1.
