# Supplementary material for: Chicken eEF1α is a Critical Factor for the Polymerase Complex Activity of Very Virulent Infectious Bursal Disease Virus
Source: Viruses. 2020 Feb 23;12(2):249. doi: 10.3390/v12020249 (PMC7077273; doi:10.3390/v12020249)
Supplement: Supplementary file 1 [file viruses-12-00249-s001.pdf]

S Table 1. Potential host proteins identified by LC-MS/MS with proper molecular weight

Protein band No.	Reference No.	Protein/Gene names	UniquePepCount	CoverPercent(%)	MW (kDa)	PI	Moleculer function
1	tr|F1NJ08	Vimentin	28	46.3	53.21	5.13	identical protein binding
	tr|F1NMU4	Tubulin beta chain	10	21.56	50.43	4.86	structural constituent of cytoskeleton
	sp|Q5ZMA2	Pre-mRNA-processing factor 19	9	16.44	55.13	6.19	ubiquitin protein ligase activity
	tr|A0A1D5PU77	ATP synthase subunit beta	8	20.26	56.63	5.44	/
	sp|Q5ZKB9	Probable ATP-dependent RNA helicase DDX6	7	13.04	54.42	8.85	ATP binding;helicase activity
	tr|A0A1D5PEV1	CUGBP Elav-like family member 2	5	8.27	55.42	8.81	RNA binding
	tr|A0A1D5P1F1	Sphingosine kinase 1	4	8.07	54.72	8.78	sphingosine kinase activity
	tr|A0A1D5PMQ5	KRTC42L	3	5.81	54.00	4.92	structural molecule activity;
	tr|F1N8Z4	RuvB-like helicase	3	5.92	50.18	6.02	transcription coactivator activity
	tr|F1N9H4	Elongation factor 1-alpha	3	6.48	50.47	9.11	translation elongation factor activity;protein kinase binding
	sp|Q5ZMS3	Eukaryotic translation initiation factor 2 subunit gamma	2	3.39	51.08	8.54	translation initiation factor activity;GTPase activity
	tr|Q5ZM95	RCJMB04_2m14	2	5.28	53.63	5.64	RNA binding

sp|O13154	Protein kinase C and casein kinase substrate in neurons protein 2	1	1.56	51.97	5.24	cytoskeletal protein binding;phospholipid binding
tr|A0A1C9UAS5	NADH-ubiquinone oxidoreductase chain 4	1	1.31	51.12	9.56	NADH dehydrogenase (ubiquinone) activity
tr|A0A1D5P0Q1	Serine hydroxymethyl transferase	1	1.91	51.71	8.36	/
tr|A0A1D5P8M8	LRR binding FLII interacting protein 2	1	1.54	52.04	5.36	regulation of transcription
tr|A0A1D5PVC5	Uncharacterized protein	1	1.62	56.68	9	/
tr|A0A1D5PXW3	Transmembrane and coiled-coil domain family 2	1	1.46	53.21	5.82	amyloid precursor protein metabolic process
tr|A0A1L1RJ97	Basal body orientation factor 1	1	1.74	54.32	8.76	/
tr|A0A2Y9V195	Glycosylation site-binding protein	1	2.76	56.89	4.84	protein disulfide isomerase activity
tr|E1C0C2	Scm polycomb group protein like 4	1	1.62	53.44	9.51	/
tr|F1NYI3	tRNA-splicing ligase RtcB homolog	1	1.78	55.24	6.79	RNA ligase (ATP) activity;vinculin binding
tr|F1P5U6	CYP2C18	1	1.82	55.76	8.71	heme binding;iron ion binding
tr|Q5ZMN1	G3BP stress granule assembly factor 1	1	2.12	52.44	5.11	mRNA binding;
tr|A0A1X9WEL5	Elongation factor Tu	7	17.09	47.11	6.8	translation elongation factor activity

	tr|E1BTT4	Hydroxyacyl-CoA dehydrogenase trifunctional multienzyme complex subunit beta	7	13.5	50.85	9.37	acetyl-CoA C-acyltransferase activity；enoyl-CoA hydratase activity
	sp|Q5ZLK1	DDB1-and CUL4-associated factor 13	6	11.69	51.43	9.45	Ribonucleoprotein
	tr|F1NDE0	39S ribosomal protein L37, mitochondrial	5	8.97	49.89	9.59	structural constituent of ribosome
2	tr|F1NRU5	NIN1 (RPN12) binding protein 1 homolog	5	9.86	47.29	8.42	Endoribonuclease activity;metal ion binding
	tr|A0A1L1RMC2	ATP synthase subunit alpha	4	8.04	50.07	9.12	/
	tr|F1NNS	Oligosaccharyl transferase 48 kDa subunit	4	8.31	47.94	5.35	dolichyl-diphosphooligosaccharide-protein glycotransferase activity
	tr|Q6EE30	Eukaryotic translation elongation factor 1	4	8.03	49.73	6.12	translation elongation factor activity
	sp|P51913	Alpha-enolase	3	5.3	47.30	6.17	phosphopyruvate hydratase activity
	tr|E1BR36	Actin like 6A	3	7.23	47.48	5.48	chromatin binding
	tr|Q5ZLB0	DEAD-box helicase 47	3	7.28	50.24	9.32	helicase activity；nucleic acid binding
	sp|Q5ZLT7	Basic leucine zipper and W2 domain-containing protein 1	2	4.31	48.02	5.75	Transcription regulation
	tr|A0A1D5P198	Tubulin alpha chain	2	4.21	50.15	4.94	GTP binding；ubiquitin protein ligase binding

	tr|A0A1D5PX51	WD repeat-containing protein 76	2	5.08	49.25	8.41	DNA binding； enzyme binding
	sp|Q90WD0	Actin-like protein 3	1	2.87	47.42	5.61	actin filament binding;ATP binding
	tr|A0A1D5PCQ0	Amyloid beta precursor protein binding family A member 1	1	2.14	47.29	4.5	amyloid-beta binding
	tr|A0A1D5PLA1	2',3'-cyclic-nucleotide 3'-phosphodiesterase	1	3.33	47.25	9.1	2',3'-cyclic-nucleotide 3'-phosphodiesterase activity;RNA binding
	tr|E1BS56	Uncharacterized protein	1	1.88	47.74	6.71	serine-type endopeptidase inhibitor activity
	tr|F1NPA2	Proteasome 26S subunit, non-ATPase 11	1	1.66	47.41	6.01	structural molecule activity
3	sp|Q5ZMQ2	Actin, cytoplasmic 2	8	23.2	41.79	5.31	ATP binding；ubiquitin protein ligase binding
	tr|A0A1D5P694	RPL4	7	12.57	41.30	10.95	/
	tr|Q800W4	TIA1 cytotoxic granule associated RNA binding protein	5	11.29	41.29	7.6	AU-rich element binding
	tr|Q800W3	TIAR	4	8.76	42.96	8.1	DNA binding;RNA binding
	tr|A0A1L1RNL4	HNRNPKL	3	8.89	44.66	6.57	DNA binding;RNA binding
	tr|R4GFH1	CAVIN2	3	6.89	43.38	5.02	protein kinase C binding
	tr|Q8AXQ0	Translocating chain-associated membrane protein	2	4.81	43.34	9.59	SRP-dependent cotranslational protein targeting to membrane, translocation
	tr|A0A1D5PFA1	Zinc finger C2HC-type containing 1A	2	5.25	41.71	10.12	/
	tr|A0A1D5NT89	Uncharacterized protein	1	1.98	40.54	6.02	/
	tr|A0A1D5P0J2	Uncharacterized protein	1	1.28	40.12	11.81	/

	tr|E1BSZ0	1-acylglycerol-3-phosphate O-acyltransferase 5	1	2.47	42.38	9.38	1-acylglycerol-3-phosphate O-acyltransferase activity
	tr|E1BYI6	RNA pseudouridylate synthase domain containing 4	1	2.82	40.02	10.01	pseudouridine synthase activity;RNA binding
	tr|E1C4L6	Integrin subunit beta 1 binding protein 2	1	2.11	41.57	5.68	/
4	sp|P53478	Actin, cytoplasmic type 5	6	18.35	41.84	5.3	ATP binding
	tr|A0A1D5P892	SRSF6	2	5.17	40.17	11.5	pre-mRNA binding
	tr|A0A1D5NXB3	Uncharacterized protein	1	1.9	40.94	6.72	RNA binding;zinc ion binding
	tr|A0A1L1RJL4	DNA polymerase beta	1	1.79	38.43	8.91	/
	tr|B5BSG4	MHC class I alpha chain 2	1	1.97	38.82	5.71	/
5	sp|Q5ZL35	Arginine and glutamate-rich protein 1	1	2.17	33.54	10.32	/
	tr|E1BQM7	Phosducin like	1	2	34.43	4.61	positive regulation of smoothened signaling pathway
	tr|E1C8K3	Annexin	1	2.21	35.65	5.96	calcium-dependent phospholipid binding
	tr|Q8UWG7	60S ribosomal protein L6	1	2.35	33.92	10.67	structural constituent of ribosome
6	tr|Q9PTD6	Ribosomal protein S6 (Fragment)	3	8.5	28.42	10.83	structural constituent of ribosome
	sp|Q5ZJ56	60S ribosomal protein L7	1	4.07	28.78	10.83	structural constituent of ribosome

	tr|A0A1D5PDK5	Transgelin 2	1	3.61	27.23	9.73	epithelial cell differentiation
	tr|A0A1D5PNE4	60S ribosomal protein L7a	1	3.08	29.33	10.48	/
	tr|Q5ZJC1	RCJMB04_19g1	1	5.33	26.86	9.68	structural constituent of ribosome
